# Valor do ^18^F-FDG PET/CT no Diagnóstico e Avaliação de Resposta ao Tratamento da Miocardite Lúpica

**DOI:** 10.36660/abc.20210523

**Published:** 2022-06-06

**Authors:** Alvaro M. Perazzo, Larissa G. F. Andrade, Leonardo G. A. Venancio, Pedro Alves da Cruz Gouveia, Mariana Feitosa Ramalho Galvão, Esdras M. Lins, Fernando Moraes, Simone Cristina Soares Brandão

**Affiliations:** 1 Divisão de Cirurgia Cardiovascular do Pronto-Socorro Cardiológico de Pernambuco - PROCAPE Recife PE Brasil Divisão de Cirurgia Cardiovascular do Pronto-Socorro Cardiológico de Pernambuco - PROCAPE, Recife, PE – Brasil; 2 Universidade de Pernambuco - UPE Recife PE Brasil Universidade de Pernambuco - UPE, Recife, PE – Brasil; 3 Universidade Federal de Pernambuco - UFPE Departamento de Cirurgia Recife PE Brasil Departamento de Cirurgia - Universidade Federal de Pernambuco - UFPE, Recife, PE – Brasil; 4 Universidade Federal de Pernambuco - UFPE Programa de Pós-Graduação em Cirurgia Recife PE Brasil Programa de Pós-Graduação em Cirurgia da Universidade Federal de Pernambuco - UFPE, Recife, PE – Brasil; 5 Universidade Federal de Pernambuco - UFPE Disciplina de Cirurgia Cardio-Torácica Recife PE Brasil Disciplina de Cirurgia Cardio-Torácica, Universidade Federal de Pernambuco - UFPE, Recife, PE – Brasil; 6 Hospital Universitário Oswaldo Cruz Departamento de Clínica Médica Recife PE Brasil Departamento de Clínica Médica do Hospital Universitário Oswaldo Cruz, Recife, PE - Brasil; 7 Universidade Federal de Pernambuco - UFPE Departamento de Cirurgia Vascular Recife PE Brasil Departamento de Cirurgia Vascular, Universidade Federal de Pernambuco - UFPE, Recife, PE – Brasil; 8 Universidade Federal de Pernambuco - UFPE Departamento de Medicina Nuclear Recife PE Brasil Departamento de Medicina Nuclear, Universidade Federal de Pernambuco - UFPE, Recife, PE – Brasil

**Keywords:** Lupus Eritematoso Sistêmico/complicações, Miocardite Lúpica, Diagnóstico por imagem/métodos, Tomografia Computadorizada por Emissão de Pósitrons Tomografia/métodos, Imunossupressores/uso terapêutico

O Lúpus Eritematoso Sistêmico (LES) é uma doença autoimune com um grande espectro de manifestações clínicas. Dentre os órgãos afetados, o sistema cardiovascular tem importância clínica relevante por estar associado a maior mortalidade nestes pacientes. O coração pode ser afetado em quaisquer de suas estruturas, sendo a miocardite lúpica um grande desafio diagnóstico na prática clínica.^1^

Exames não invasivos como eletrocardiograma e ecocardiograma não são sensíveis ou específicos o suficiente para esse diagnóstico. A ressonância magnética cardíaca (RMC) é a modalidade de imagem preferida para diagnóstico de miocardite, porém tem suas contraindicações, tais como portadores de implantes metálicos ou o uso de gadolínio na doença renal crônica.

A biópsia miocárdica, apesar de ser considerada o padrão-ouro, tem a grande desvantagem de ser um procedimento invasivo com riscos inerentes ao procedimento.^2^ Desta forma, tem-se estudado alternativas diagnósticas com maior sensibilidade, especificidade e com menor risco para o paciente.

O uso da tomografia por emissão de pósitrons associado a tomografia computadorizada com fluorodeoxiglicose (^18^F-FDG PET/CT) surge como um novo método de imagem para avaliação de processos inflamatórios em doenças reumatológicas, incluindo o LES.^3 , 4^ O ^18^F-FDG PET/CT combina a técnica da medicina nuclear com imagens de tomografia computadorizada.

Embora o miocárdio possa captar glicose como substrato energético, na investigação de processos inflamatórios cardíacos, o preparo com jejum de no mínimo 12h, dieta pobre em carboidratos, rica em gordura e uso de heparina 15 minutos antes da injeção do ^18^F-FDG suprime a captação fisiológica de glicose pelos cardiomiócitos. Assim, se visualizarmos captação cardíaca de ^18^F-FDG, infere-se captação por células inflamatórias, uma vez que elas não sofrem interferência na captação de glicose com este preparo.^5^ Há poucos trabalhos associando o uso do ^18^F-FDG PET/CT ao diagnóstico e acompanhamento da miocardite lúpica.^3 , 4^ A imagem deste caso é de uma paciente do sexo feminino, 16 anos, internada com quadro de febre persistente, perda de peso importante, tosse, edema e atraso menstrual. Iniciou investigação para diversas doenças infecciosas incluindo a pericardite tuberculosa, assim como, para doenças autoimunes. Dentre os exames realizados, o ecodopplercardiograma transtorácico evidenciou déficit biventricular, hipertensão arterial pulmonar e insuficiência mitral importante. No decorrer da investigação diagnóstica, devido a disfunção renal, optou-se pela realização do ^18^F-FDG PET/CT. Após a realização deste exame, que mostrou hipercaptação cardíaca, de grau acentuado e difuso de FDG ( Figura 1 ), aventou-se a possibilidade de miocardite lúpica, que diante de todo o contexto clínico foi posteriormente confirmada pelos testes sorológicos. A paciente foi tratada com imunossupressores (Metilprednisolona e Micofenolato de mofetila) e após 2 meses repetiu o exame mostrando regressão completa da captação pelo miocárdio ( Figura 2 ). Considerando o caso clínico em questão e mediante revisão de literatura, sugere-se que o uso do ^18^F-FDG PET/CT pode ser útil e promissor no diagnóstico e seguimento de pacientes com miocardite lúpica. Mais estudos clínicos destinados a avaliar este método diagnóstico nessa população serão necessários.

**Figura 1:**
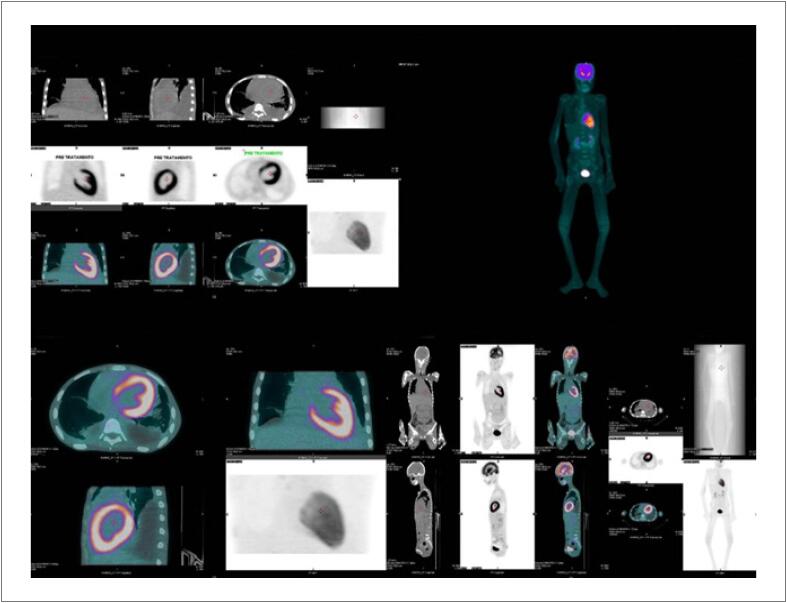
^18^F-FDG PET/CT na miocardite lúpica pré-tratamento: Observa-se intensa captação difusa de 18F-FDG no ventrículo esquerdo inferindo quadro de miocardite

**Figura 2:**
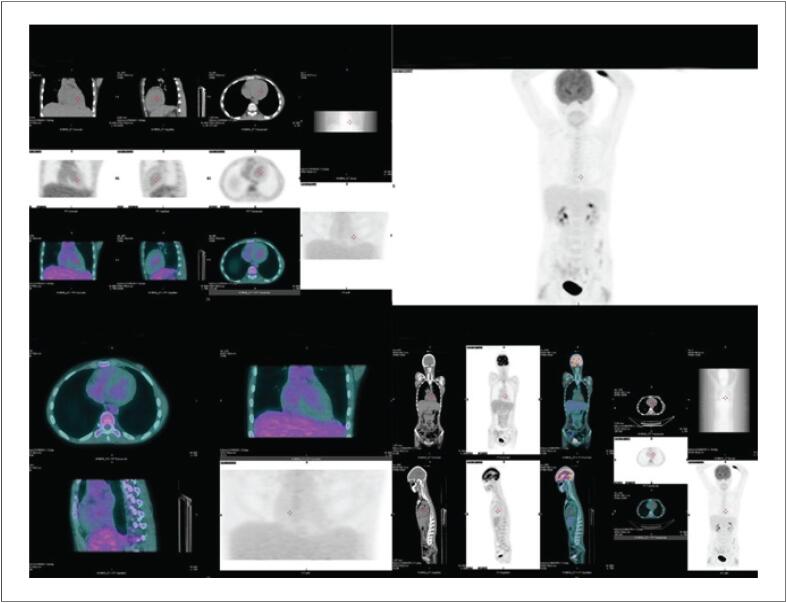
^18^F-FDG PET-CT na miocardite lúpica pós-tratamento: Observa-se regressão completa da captação após 2 meses de tratamento com imunossupressor.

## References

[1] Doria A, Laccarino L, Sarzi-Puttini P, Atzeni F, Turriel M, Petri M. Cardiac involvement in systemic lupus erythematosus. Lupus. 2005;14(9):683-6. doi: 10.1191/0961203305lu2200oa. 10.1191/0961203305lu2200oa 16218467

[2] Cooper LT. Myocarditis. N Engl J Med. 2009;360(15):1526-38. doi: 10.1056/NEJMra0800028. 10.1056/NEJMra0800028 PMC5814110 19357408

[3] Alchammas J, Al-Faham Z, Roumayah Y, Wong OCY. The evaluation of lupus myocarditis with 13N-Ammonia and 18F-FDG PET. J Nucl Med Technol. 2016;44(3):210-1. doi: 10.2967/jnmt.115.165639. 10.2967/jnmt.115.165639 26769599

[4] Perel-Winkler A, Bokhari S, Perez-Recio T, Zartoshti A, Askanase A, Geraldino-Pardilla A. Myocarditis in systemic lupus erythematosus diagnosed by 18 F-fluorodeoxyglucose positron emission tomography. Lupus Sci Med. 2018; 5(1):e000265. doi: 10.1136/lupus-2018-000265. 10.1136/lupus-2018-000265 PMC6069920 30094040

[5] Al-Fahan Z, Jolepalem P, Wong CO. The evaluation of cardiac sarcoidosis with 18F-FDG PET scan. J Nucl Med Technol. 2016; 44(2):92-3. DOI: 10.2967/jnmt.115.158857 10.2967/jnmt.115.158857 26271805

