# Supplementary material for: Valor do 18F-FDG PET/CT no Diagnóstico e Avaliação de Resposta ao Tratamento da Miocardite Lúpica
Source: Arq Bras Cardiol. 2022 Jun 6;118(6):1150–2. [Article in Portuguese] doi: 10.36660/abc.20210523 (PMC9345146; doi:10.36660/abc.20210523)
Supplement: Translation

# Translation: Value of^18^F-FDG PET/CT in the Diagnosis and Assessment of Response to Treatment of Lupus Myocarditis

**Authors:** Alvaro M. Perazzo, Larissa G. F. Andrade, Leonardo G. A. Venancio, Pedro Alves da Cruz Gouveia, Mariana Feitosa Ramalho Galvão, Esdras M. Lins, Fernando Moraes, Simone Cristina Soares Brandão

**Affiliations:** 1 Recife PE Brazil Divisão de Cirurgia Cardiovascular do Pronto-Socorro Cardiológico de Pernambuco - PROCAPE, Recife, PE – Brazil; 2 Recife PE Brazil Universidade de Pernambuco - UPE, Recife, PE – Brazil; 3 Recife PE Brazil Departamento de Cirurgia - Universidade Federal de Pernambuco - UFPE, Recife, PE – Brazil; 4 Recife PE Brazil Programa de Pós-Graduação em Cirurgia da Universidade Federal de Pernambuco - UFPE, Recife, PE – Brazil; 5 Recife PE Brazil Disciplina de Cirurgia Cardio-Torácica, Universidade Federal de Pernambuco - UFPE, Recife, PE – Brazil; 6 Recife PE Brazil Departamento de Clínica Médica do Hospital Universitário Oswaldo Cruz, Recife, PE - Brazil; 7 Recife PE Brazil Departamento de Cirurgia Vascular, Universidade Federal de Pernambuco - UFPE, Recife, PE – Brazil; 8 Recife PE Brazil Departamento de Medicina Nuclear, Universidade Federal de Pernambuco - UFPE, Recife, PE – Brazil

**Keywords:** Lupus Erythematosus, Systemic/complications, Lupus Myocarditis, Diagnostic, Imaging, Tomography Computed Emission Positrons Tomography/methods, Immunosupressive Agents/therapeutic use

Systemic Lupus Erythematosus (SLE) is an autoimmune disease with a wide spectrum of clinical manifestations. The cardiovascular system has relevant clinical importance among the affected organs because it is associated with higher mortality in these patients. The heart can be affected in any structure, and lupus myocarditis is a major diagnostic challenge in clinical practice.^1^

Non-invasive tests such as electrocardiograms and echocardiograms are not sensitive or specific enough for this diagnosis. Cardiac magnetic resonance imaging (CMR) is the preferred imaging modality for diagnosing myocarditis, but it has contraindications, such as patients with metallic implants or using gadolinium in chronic kidney disease.

Despite being considered the gold standard, myocardial biopsy has the great disadvantage of being an invasive procedure with inherent risks.^2^Thus, diagnostic alternatives with greater sensitivity, specificity, and less risk to the patient have been studied. The use of positron emission tomography associated with computed tomography with fluorodeoxyglucose (^18^F-FDG PET/CT) emerges as a new imaging method for evaluating inflammatory processes in rheumatologic diseases, including SLE.^3 , 4^^18^F-FDG PET/CT combines the technique of nuclear medicine with computed tomography imaging.

Although the myocardium can capture glucose as an energy substrate, in the investigation of cardiac inflammatory processes, the preparation with fasting of at least 12 hours, a low-carbohydrate diet, fat and use of heparin 15 minutes before injection of^18^F-FDG suppresses physiological glucose uptake by cardiomyocytes. Thus, if we visualize cardiac uptake of^18^F-FDG, uptake by inflammatory cells is inferred since they do not suffer interference in glucose uptake with this preparation.^5^Few studies associate^18^F-FDG PET/CT with the diagnosis and follow-up of lupus myocarditis.^3 , 4^The image of this case is of a female patient, 16 years old, hospitalized with persistent fever, significant weight loss, cough, edema, and menstrual delay. She initiated research for several infectious diseases, including tuberculous pericarditis and autoimmune diseases. Among the tests performed, the transthoracic echocardiogram showed a biventricular deficit, pulmonary arterial hypertension, and severe mitral regurgitation. It was decided to perform 18F-FDG PET/CT during the diagnostic investigation due to renal dysfunction. After performing this examination, which showed a marked and diffuse cardiac hyperuptake of FDG ( Figure 1 ), the possibility of lupus myocarditis was raised, which was later confirmed by serological tests, given the entire clinical context. The patient was treated with immunosuppressants (Methylprednisolone and mycophenolate mofetil), and after 2 months, the exam was repeated, showing complete regression of myocardial uptake ( Figure 2 ). Considering the clinical case in question and a literature review, it is suggested that the use of^18^F-FDG PET/CT may be useful and promising in the diagnosis and follow-up of patients with lupus myocarditis.

**Figure 1:** ^18^F-FDG PET/CT in pre-treatment lupus myocarditis: There is intense diffuse uptake of^18^F-FDG in the left ventricle, suggesting myocarditis.

**Figure 2:** ^18^F-FDG PET-CT in post-treatment lupus myocarditis: Complete regression of uptake is observed after 2 months of immunosuppressant treatment.
